# Regorafenib suppresses colon tumorigenesis and the generation of drug resistant cancer stem-like cells via modulation of miR-34a associated signaling

**DOI:** 10.1186/s13046-018-0836-x

**Published:** 2018-07-13

**Authors:** Mao-Hua Cai, Xiao-Gang Xu, Shi-Li Yan, Ze Sun, Yin Ying, Bai-Kui Wang, Yue-Xing Tu

**Affiliations:** 1 Department of General Surgery, Chun’an First People’s Hospital (Zhejiang Provincial People’s Hospital Chun’an Branch), Hangzhou, 311700 Zhejiang Province China; 2 Key Laboratory of Molecular Animal Nutrition of Ministry of Education, Institute of Feed Science, College of Animal Sciences, Zhejiang University, Hangzhou, 310029 Zhejiang Province China; 3 Zhejiang Academy of Traditional Chinese Medicine, Hangzhou, 310007 Zhejiang Province China; 4 Department of Critical Care Medicine, Zhejiang Provincial People’s Hospital, People’s Hospital of Hangzhou Medical College, No. 158 Shangtang Road, Hangzhou, 310014 China

**Keywords:** 5-fluouracil resistant colon cancer, Cancer stem-like cells, Regorefanib, WNT/β-catenin signaling and miR-34a

## Abstract

**Background:**

Colorectal cancer (CRC) is one of the most prevalent malignancies in the world and developed drug resistance has represented one of the most challenging tasks for management. The current therapeutic regimens may select and enrich cancer stem-like cells (CSCs) resulting in the increased resistance against treatment, metastatic potential and mortality. Regorafenib is a multi-kinase inhibitor, an FDA-approved last-of-line treatment for patients with chemo-refractory metastatic CRC. However, regorafenib’s potential effects on CSCs have not been fully elucidated.

**Methods:**

Here, we developed two 5-FU resistant CRC cell lines, HCT-116R and DLD-1R and showed the increased CSCs characteristics such as increased side-population cells, tumor sphere formation and expression of stemness markers. These cell lines and CSCs properties were used for evaluating the potential of regorafenib in suppressing CSCs.

**Results:**

We showed that regorafenib treatment decreased the stemness phenotypes including tumor sphere formation, and side-population, of both HCT-116R and DLD-1R cells. Additionally, regorafenib suppressed the cell viability in both cell lines synergistically with 5-FU. In vivo, the combination of regorafenib and 5-FU significantly suppressed the tumorigenesis and stemness markers of 5-FU resistant DLD-1R. Mechanistically, regorafenib-mediated effects were associated with the induction of tumor suppressor miR-34a and suppression of WNT/β-catenin signaling. Our findings demonstrated that regorafenib treatment was associated with the increased level of miR-34a, resulting in reversing drug resistance and cancer-initiating cell phenotypes by degrading WNT/β-catenin in CRC.

**Conclusion:**

Regorafenib might be a potential drug for colon cancer stem-like cells and it should be investigated in future clinical trials.

**Electronic supplementary material:**

The online version of this article (10.1186/s13046-018-0836-x) contains supplementary material, which is available to authorized users.

## Background

Colorectal cancer (CRC) has consistently ranked the top 5 most deadly malignancies in the developed countries due to its highly metastatic potential and the resistance against treatments. Despite the advance in the development of chemo- and targeted therapeutic agents, the mortality and the recurrence rates remain high in the advanced stage patients [1]. Accumulating evidence demonstrates that these currently available agents select for and/or enrich the cancer stem-like cells [2] leading to the eventual treatment failure. Thus, the identification of alternative or novel agents is urgently required.

Regorafenib is an approved multiple kinase inhibitor for the patients with metastatic colon cancer and failed to respond to currently available chemotherapeutic agent [3]. Regorafenib has been shown to inhibit tumorigenesis both in vitro and in vivo via downregulating RAF/MEK/ERK signaling in different cancer types including colon, breast and renal cell carcinoma [4]. Interestingly, *KRAS*, *BRAF* or *PIK3CA* status was not found to be predictive for the positive regorafenib responders in the clinical settings [5]. Thus, the precise patient population who may be benefited from regorafenib and the underlying mechanism of actions still require further investigation.

miR-34a is highly expressed in normal tissues and commonly repressed in carcinoma such as prostate cancer, breast cancer, ovarian cancer and non-small cell lung cancer. Moreover, previous studies demonstrated that ectopic expression of miR-34a suppressed cell proliferation, migration and invasion in various cancer cells, which could also contribute to drug resistance in breast cancer by targeting a variety of oncogenes [6]. However, the role and mechanism of miR-34a in the regulation of colon cancer stem-like cells is far from being completely elucidated at present.

It was our goal to investigate the potential efficacy of regorafenib on cancer stem-like cells in this study. We first established two colon cancer cell lines resistant to fluorouracil by acclimatization. These 5-FU resistant colon cancer cells exhibited enhanced tumorigenic phenotypes including CD44+ and side-population cells, increased colony and tumor sphere formation. These observations were associated with the elevated expression of stemness markers such as Nothc1, WNT1 and β-catenin. Importantly, we showed that the treatment of regorafenib in these 5-FU resistant cancer cells suppressed the aforementioned tumorigenic phenotypes and stemness markers. The combination of regorafenib and 5-FU synergistically suppressed colon cancer viability both in vitro and in vivo. Finally, regorafenib treatment was mechanistically associated with the increased level of a tumor suppressor, miR-34a. Thus, this study provides novel and important mechanistic explanations underlying regorafenib’s ability to treat chemo-refractory colon cancer and the combination of regorafenib and 5-FU regimen may provide an improved treatment efficacy.

## Methods

### Chemicals and reagents

Regorafenib (BAY 73–4506, catalog No. S1178) and Fluorouracil (5-FU, catalog No. S1209) were purchased from SelleckChem. The primary and secondary antibodies used for western blotting and immunohistochemical experiments were all purchased from Cell Signaling Technology unless otherwise specified.

### Generation of 5-FU resistant cell lines

Human colon cancer cell lines, HCT-116 and DLD-1 were purchased from the American Type Culture Collection (ATCC, Manassas, VA) and the cells were maintained under the conditions accordingly. To generate 5-FU resistant cells, both HCT-116 and DLD-1 cells were initially exposed to 5-FU (5 μM 72 h) and the survived cells were subsequently passaged and maintained under the same culture conditions for at least 20 more passages. The resultant 5-FU-acclimatized cells were termed HCT-116R and DLD-1R (R as 5-FU resistant line).

### Side population (SP) and tumor sphere assays

We performed the side population (SP) assay to identify and quantify the cancer stem-like and/or drug resistant cancer cells. SP cells are defined as a sub-population of cells with high expression of ATP-binding cassette transporters (ABCG2) and the ability to exclude Hoechst 33,342 nuclear dye [7]. We used FACSAria™ technology platform to determine and compare the SP cells in HCT-116, HCT-116R, DLD-1 and DLD-1R cells. Cells were first labeled with Hoechst 33342 dye (2.5 μg/mL) for 30 min at 37 °C. The control cells were treated with verapamil (50 μM, Sigma-Aldrich). Propidium iodine (PI) 1 μg/mL served to identify dead cells. After identification and cell sorting, SP cells were cultured under stem cell conditions: serum-free of HEScGRO medium, N2 supplement (Invitrogen, Carlsbad, CA), 10 ng/mL human recombinant bFGF (Invitrogen), and 10 ng/mL EGF (Invitrogen) in ultra-low attachment CoStar plates (Corning, NY). Tumor spheres were measured and those ≥ 200 μm were counted as a tumor sphere forming unit. The data calculated for the number and size of the tumor spheres is the average of three independent experiments.

### Cell viability test

Sulforhodamine B (SRB) dye (Sigma-Aldrich, Chemie GmbH, Munich, Germany) was used to test the effects of selective inhibitors on cell growth and viability of SP cells. The regorafenib were dissolved in dimethyl sulfoxide (DMSO) before diluting with growth medium to a final DMSO concentration of 0.05%. The HCT-116R and DLD-1R cells were seeded into 96 well plates in growth medium at 3000 cells/well. After 24 h the medium was replaced with fresh growth medium containing the regorafenib. The cells were incubated for another 48 h. The cells were fixed with trichloroacetic acid (TCA) by gently adding 50 μL TCA (50%) to each well to a final TCA concentration of 10% with subsequent incubation for 1 h at 4 °C. The plates were then washed 5 times with tap water and air dried. The dried plates were stained with 100 μL of 0.4% (*w*/*v*) SRB prepared in 1% (*v*/v) acetic acid for 10 min at room temperature. The plates were rinsed quickly 4 times with 1% acetic acid to remove unbound dye, and then air dried until no moisture was visible. The bound dye was solubilized in 20 mM Tris-base (100 μL/well) for 5 min on a shaker. Optical densities were read on a microplate reader (Molecular Devices, Sunnyvale, CA) at 562 nm.

### SDS-PAGE and western blotting

HCT-116R and DLD-1R cells were lysed and prepared using ReadyPrep Protein Extraction Kit (Bio-Rad, Hercules, CA) according to the vendor’s instructions. Total cell lysates (50 μg) were separated by a 10% SDS-PAGE and transferred to a PVDF (polyvinylidene fluoride) membrane via BioRad Protean III system. The blots were then blocked with 5% skim milk in PBST and incubated with primary antibodies overnight at 4 °C. All primary antibodies: β-catenin, Nanog, p65/RelA (NF-kB), Bax, p-mTOR, Wnt1, STAT3, Notch1, c-Myc, vimentin and β-actin (as loading control) were purchased from Cell Signaling unless otherwise specified. The membranes were then incubated with peroxidase-conjugated secondary antibody at room temperature for at least 1 h. Blots were washed 3 times with PBST. Signals were then detected using enhanced chemiluminescence kit and visualized using the BioSpectrum Imaging System (UVP, Upland, CA).

### In vivo studies

All animal studies were performed strictly under the University’s animal experiment protocol. In the comparative tumorigenesis experiment, DLD-1 and DLD-1R cells were injected into the flank for NOD/SCID mice (1 × 10^6^ cells/mouse; *N* = 5/group). In the drug treatment experiments, DLD-1R cells (1 × 10^6^ cells/mouse; N = 5/group) were injected subcutaneously into the flank of NOD/SDCI mice. The treatments started when the tumor size reached approximately 100 mm^3^ determined by a caliper. Mice were randomly divided into 4 groups: Control (sham injection), 5-FU alone (30 mg/kg, 2 times/week), regorafenib only (10 mg/kg, 5 times/week), 5-FU plus regorafenib combination (5-FU, 30 mg/kg, 2 times/week and Regorafenib, 10 mg/kg, 5 times/week, respectively). Both drugs were given intraperitoneally. The change in tumor burden was expressed as a ratio (fold change in mm^3^) as compared to the initial tumor volume. Mice were humanely sacrificed post experiment and tumor samples were collected for further analyses.

### Immunohistochemistry

Tumor tissues were fixed in 10% formalin and embedded in paraffin. Serial sections of the embedded specimens were deparaffinized and then rehydrated in a graduated fashion and stained with hematoxylin and eosin (H&E). For immunohistochemical staining, the deparaffinized slides were subjected to antigen retrieval and probed with anti-Wnt1 (1:100), anti-β-catenin (1:200), or anti-Bax (1:100) antibodies. Slides were washed and incubated with biotinylated link universal antiserum, followed by Horseradish Peroxidase Streptavidin (HRP Streptavidin). The slides were rinsed, and color was developed using 3,3-diaminobenzidine hydrochloride as a chromogen. Finally, sections were rinsed in distilled water, counterstained with Mayer’s hematoxylin, and mounted with DPX mounting medium for evaluation. Pictures were captured with a Photometrics CoolSnap CF color camera (Nikon).

### Statistical analysis

Each experiment was performed in triplicates. The results were expressed as means ± SD. The significant difference between control and experimental groups was analyzed using t-test. (*, *P*<0.05; **, *P*<0.01).

## Results

### Chronic exposure to 5-FU selects for cancer stem-like properties

**Fig. 1:**
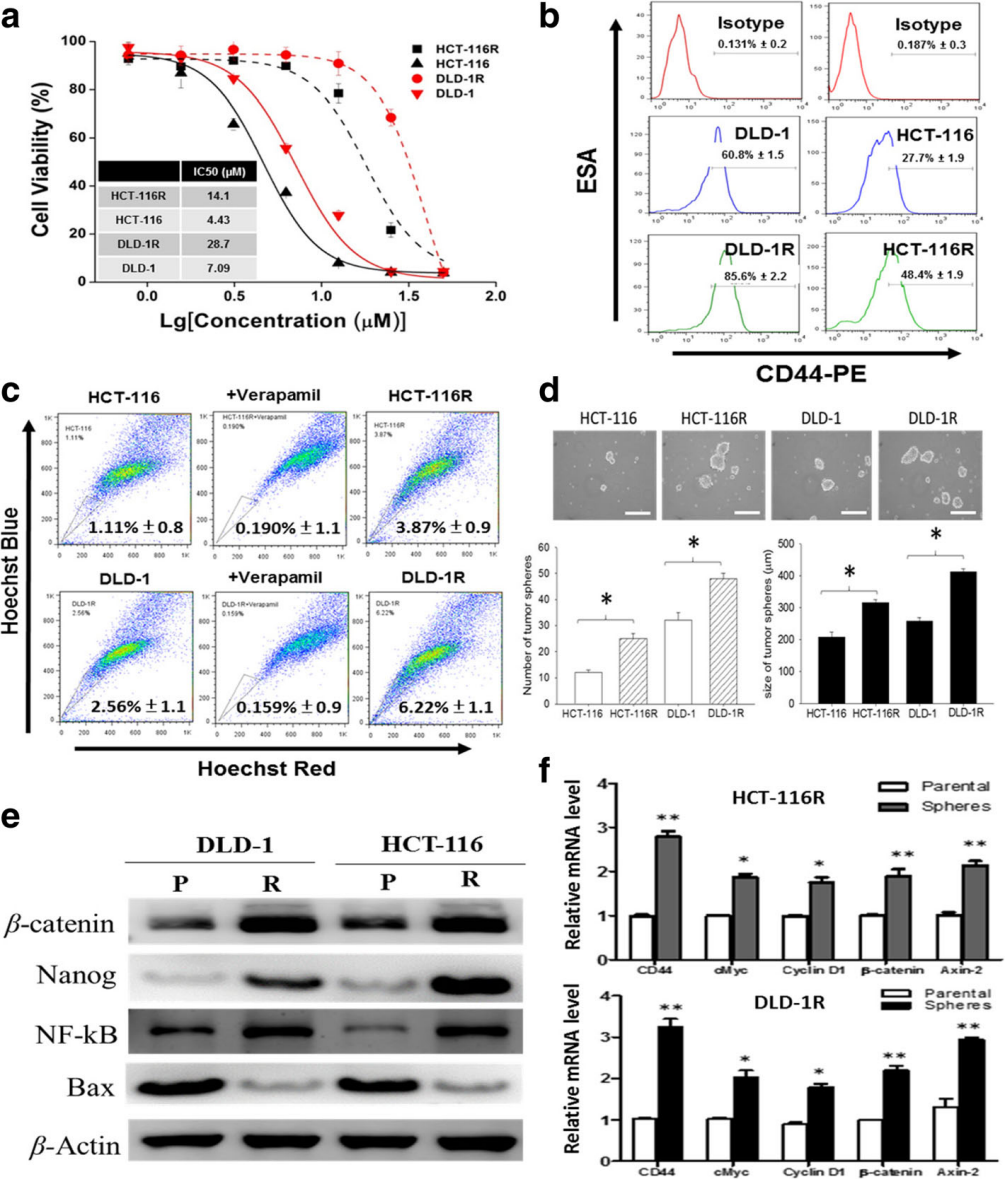
Chemo-resistant colon cancer cells exhibited increased cancer stem cell phenotypes. **a** Establishment of 5-FU resistant (designated as R) colon cancer cell lines. Both HCT116R and DLD-1R cells exhibited a significantly increased resistance against 5-FU as compared to their non-acclimatized counterparts. **b** Flow cytometric analysis of respectively. The CD44+ cell population cells were increased by approximately 12.4 and 21.8% in HCT-116 and DLD-1 cells respectively. **c** The percentage of SP cells in both cell lines were also increased, approximately 2.64% in HCT-116 and 4.31% in DLD-1, respectively. **d** Comparative tumor sphere formation essay. There was an average of 2.3-fold and 3.2-fold increase in the number of spheres (diameter ≥ 200 μm is considered as a sphere) formed in the HCT-116R and DLD-1R cells as compared to their naïve counterparts. **e** Comparative western blot analysis between the parental (P) and 5-FU acclimatized CRC cells. A significant increased expression in NF-kB, Notch1, WNT1 in the 5-FU acclimatized HCT-116 (R) and DLD-1 (R) cells. **f** Comparative real-time PCR analysis between parental colon cancer cells and their spheroid counterparts. Oncogenic markers, cMyc, cyclin D1 and stem cell associated markers, CD44, β-catenin and Axin-2 were all significantly up-regulated in the spheroids as compared to their parental counterparts. * *p* < 0.05. **p < 0.01

Colon cancer cell lines HCT-116 and DLD-1 were subjected to chronic exposure to 5-FU and the resultant cells (termed HCT-116R and DLD-1R) were collected and characterized. We found after a 6-month low-dose 5-FU exposure yielded cells with cancer stem-like properties. First, both cell lines showed increased resistance against 5-FU in cell viability assay (Fig. 1a). Comparatively, the IC_50_ values of HCT116 and HCT116R were 4.43 versus 14.1 μM respectively, approximately a 3-fold difference. Second, using flow cytometric analysis, we demonstrated that HCT116R and DLD-1R cells contained a significantly higher percentage of CD44+ cells as compared to their naïve counterparts (Fig. 1b). In the case of HCT116R, an approximately 21% increase in the CD44+ cells. Similarly, HCT116R and DLD-1R cells showed a significantly increased percentage of side-population (SP) cells (Fig. 1c). Consistently, the ability to form tumor spheres was significantly enhanced in both HCT116R and DLD-1R cells, as reflected by the increased number of spheres generated under the serum-free culture conditions. In addition, the size of the tumor spheres was also significantly increased in both HCT116R and DLD-1R cells. The average diameter of the spheres formed by HCT-116 and DLD-1 was 208 ± 15 μm and 258 ± 11 μm compared with 315 ± 10 μm and 412 ± 10 μm for the spheres formed by HCT-116R and DLD-1R (*p* < 0.05) (Fig. 1d). Molecularly, the observed inclination towards the cancer stem-like properties was associated with the increased expression level of Notch1, WNT1 (both markers of stemness), NF-κB and Vimentin (Fig. 1e). In agreement, our real-time PCR analysis demonstrated that the mRNA level of oncogenic genes including cMyc, cyclin D1 and cancer stem cell associated markers, CD44, β-catenin and Axin-2 was all up-regulated in the spheres as compared to their parental counterparts (Fig. 1f).

### DLD-1R cells demonstrated an increased tumorigenic ability in vivo

**Fig. 2:**
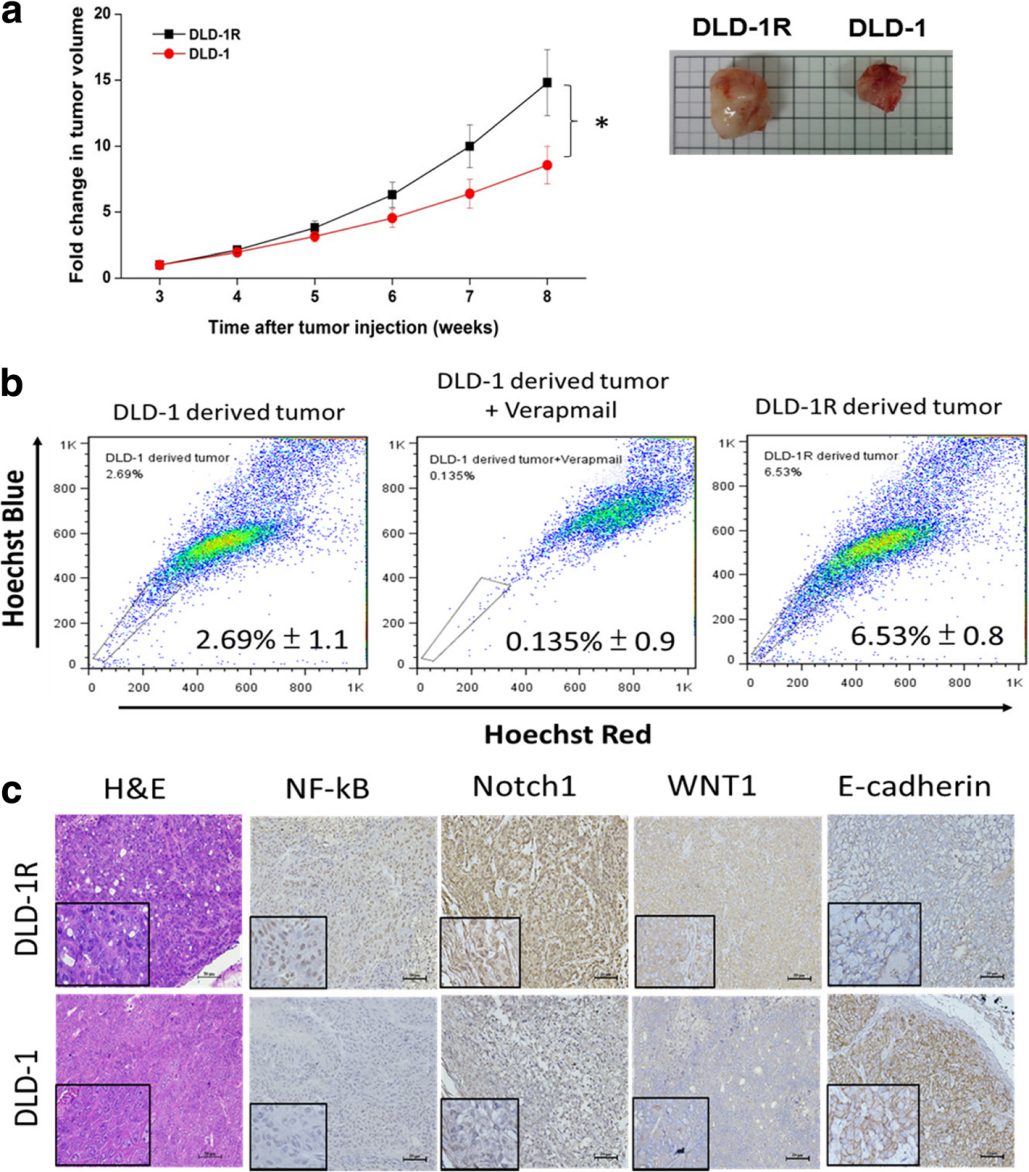
DLD-1R cells exhibits increased tumor initiating ability in vivo. **a** Comparative in vivo tumor initiating ability between DLD-1R versus naïve DLD-1 counterparts. NOD/SCID mice were injected with the same number of DLD-1R and naïve cells (1 × 10^6^ cells/injection) and tumor growth was monitored over time. DLD-1R bearing mice showed a significantly higher tumor burden as compared to their naïve counterparts. Photographs of harvested tumor samples are shown. **b** Representative FACS analysis demonstrated the Increased percentage of SP cells in tumor harvested from DLD-1R mice comparing to DLD-1 naïve samples. **c** Comparative immunohistochemical analysis showing an elevated expression of WNT1, NF-kB, Notch-1 and decreased expression of E-cadherin. The inserts show the magnified view of the corresponding sections

Next, we set out to determine if the increased stem-like characteristics could be verified in vivo by using subcutaneous DLD-1R tumor xenograft mouse model. Comparatively, mice received DLD-1R cells showed a significantly larger tumor burden than their DLD-1 parental counterparts (Fig. 2a). The tumor cells were harvested and undergone comparative stemness analysis. We found that harvested DLD-1R tumor cells exhibited approximately twice as many side-population (SP) cells as compared to their parental DLD-1 counterparts (6.53% versus 2.69% respectively, Fig. 2b). In support, the immunohistochemical analysis of the tumor samples showed a higher expression level of NF-κB, Notch1 and WNT1 in DLD-1R tumors than its DLD-1 parental samples; a reverse observation was made in E-cadherin expression (Fig. 2c).

### Regorafenib treatment suppressed colon cancer stem-like phenotypes

**Fig. 3:**
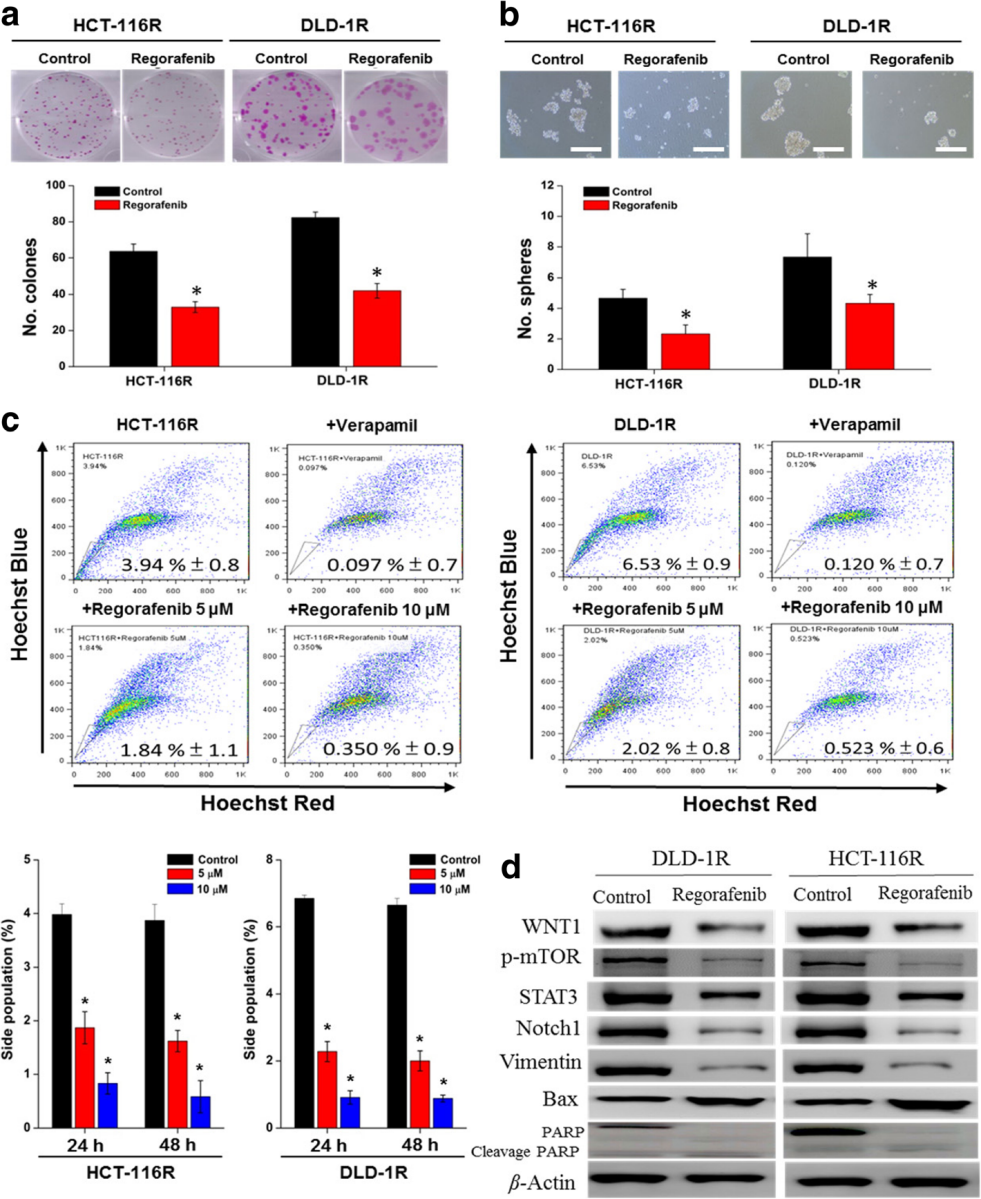
Regorefenib suppresses stemness phenotypes in DLD-1R cells. Regorefenib treatment significantly suppressed colony formation (**a**), tumor sphere formation (**b**) and the percentage of SP cells in DLD-1R and HCT-116R cells (**c**). **d** Western blot analysis shows the decreased protein expression of stemness (WNT1, Notch1) and oncogenic (phosphorylated form of mTOR, STAT3 and vimentin) markers while an increased level of the pro-apoptotic markers, Bax and PARP (cleaved PARP). *p < 0.05

Regorafenib has been an FDA-approved drug for treating metastatic colon cancer since 2012, however, its potential usage against drug-resistant colon cancer cells has not been fully explored. Here, we took advantage of our established 5-FU resistant HCT-116R and DLD-1R cell lines to examine regorafenib’s efficacy against these cells. We found that regorafenib significantly suppressed the colon at 2 μM in both HCT-116R and DLD-1R cells (Fig. 3a). Notably, at this concentration, little or no colony was able to form in both parental HCT-116 and DLD-1 cells. Similarly, regorafenib treatment (at a slightly higher concentration, 5 μM) was able to significantly suppress the generation of tumor spheres in both HCT-116R and DLD-1R cells (Fig. 3b), with DLD-1R being more resistant. Consistently, regorafenib treatment dose-dependently (ranging from 0, 1, 5 to 10 μM) decreased the percentage of SP cells in both cell lines. For instance, regorafenib treatment dose- and time-dependently decreased the percentage of DLD-1R SP cells from approximately 6.53 to 0.523% (at 10 μM) (Fig. 3c). These observations were molecularly supported by our western blot analysis, depicting the decreased expression of WNT1, Notch1 (stemness markers), p-mTOR and STAT3 (oncogenic signaling pathway), Vimentin (mesenchymal marker) and an increased expression in Bax (pro-apoptotic marker) in regorafenib-treated HCT-116R and DLD-1R cells (Fig. 3d).

### Regorafenib-mediated stemness inhibitory effect was associated with the induction of miR-34a

**Fig. 4:**
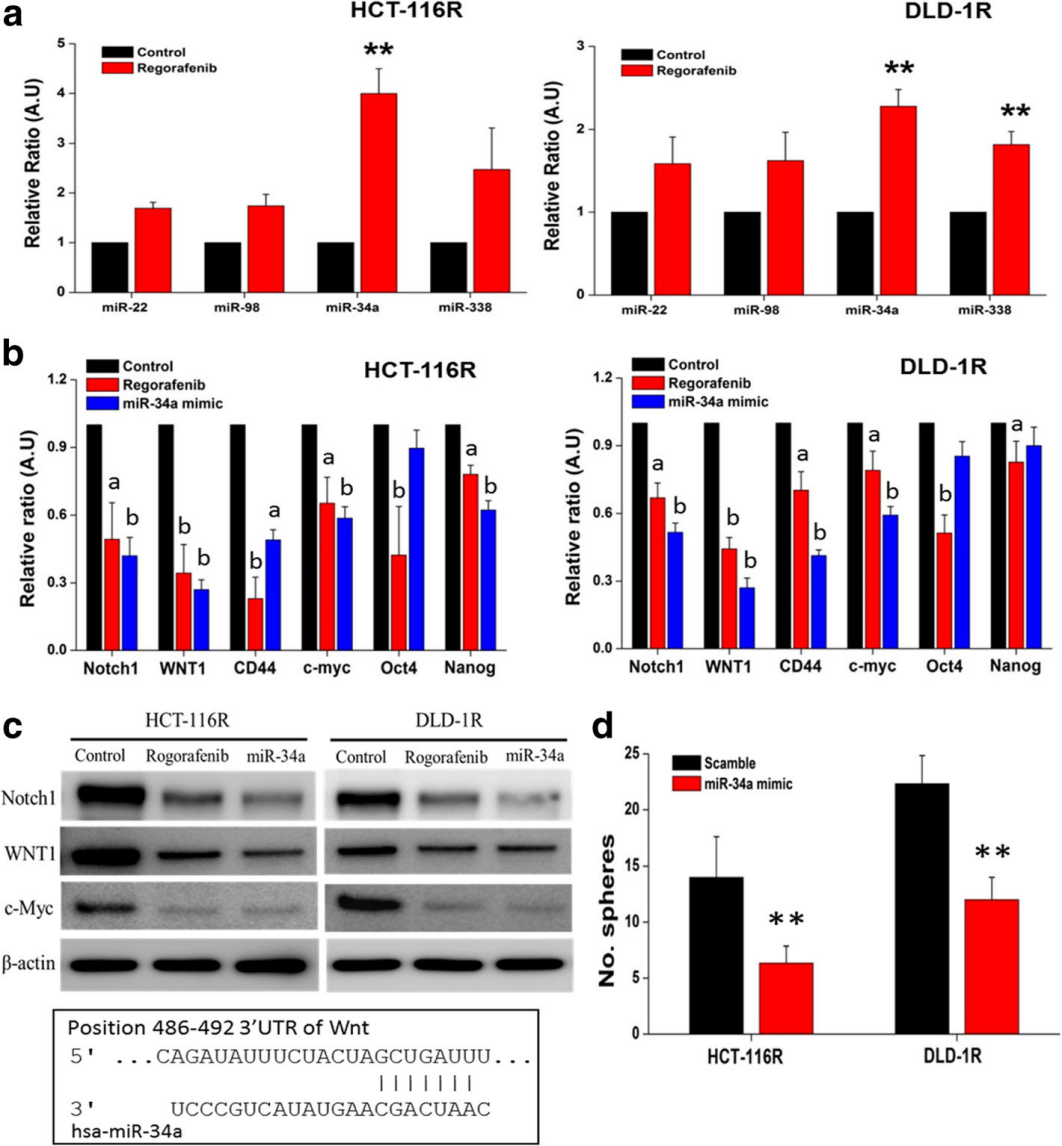
Regorafenib suppressed drug-resistant CRC cells via miR-34a induction. **a** Screening of tumor suppressor microRNAs post regorafenib treatment. Regorafenib treatment increased the level of microRNAs associated with tumor suppression. miR-34a was found to be increased most significantly (approximately 4 and 2.5-fold in HTC-116R and DLD-1R respectively). **b** Quantitative PCR analysis of stemness genes affected by regorafenib and miR-34a mimic treatment. In both HCT-116R and DLD-1R cells, stemness genes including Notch1, WNT1, CD44 and c-Myc were both suppressed by either regorafenib and exogenous miR-34a. a, *p* < 0.05; b, *p* < 0.01. **c** Comparative western blot analysis. Stemness markers including Notch1, WNT1 and c-Myc were decreased by the treatment of regorafenib and the addition of miR-34a mimic molecules. The insert demonstrates the binding of miR-34a to the 3’UTR of Wnt gene (TargetScan software). **d** Tumor sphere forming assay. Exogenous miR-34a suppressed the tumor sphere generating ability in both HCT-116R and DLD-1R cells. **, *p* < 0.01

To explore the potential mechanism of action mediated by regorafenib in suppressing the cancer stem-like phenotypes, we examined a small panel of tumor suppressor microRNAs. Among the four tumor suppressors (miR-22, miR-98, miR-34a and miR-338) suggested or shown to be associated with colon cancer, miR-34a expression level was elevated in both HCT-116R and DLD-1R cells (Fig. 4a). At the mRNA level, both regorafenib treatment or miR-34a mimic molecule was able to suppress Notch1, WNT1, CD44 and c-Myc in both cell lines (Fig. 4b). A similar observation was made in the western blot analysis, where the expression level of Notch1, WNT1 and c-Myc was decreased by either regorafenib or miR-34a mimic treatment (Fig. 4c). In support, using TargetScan software, we identified a potential binding site of miR-34a in the 3’UTR of Wnt (insert, Fig. 4c). More importantly, the addition of miR-34a mimic molecule was associated with the significantly decreased number of tumor spheres generated in both HCT-116R and DLD-1R cells (Fig. 4d). The increased miR-34a level is associated with the decreased sphere-forming ability. Western blot analysis of tumor spheres of both HCT-116R and DLD-1R treated with miR-34a mimic and inhibitor molecules. Increased miR-34a by the mimic treatment resulted in the decreased stemness markers, Notch1, Wnt1 and oncogene c-Myc. The reversal effect was observed when miR-34a inhibitor was added (Additional file 1: Figure S1).

### Regorafenib and 5-FU combination suppressed 5-FU resistant colon cancer cells

**Fig. 5:**
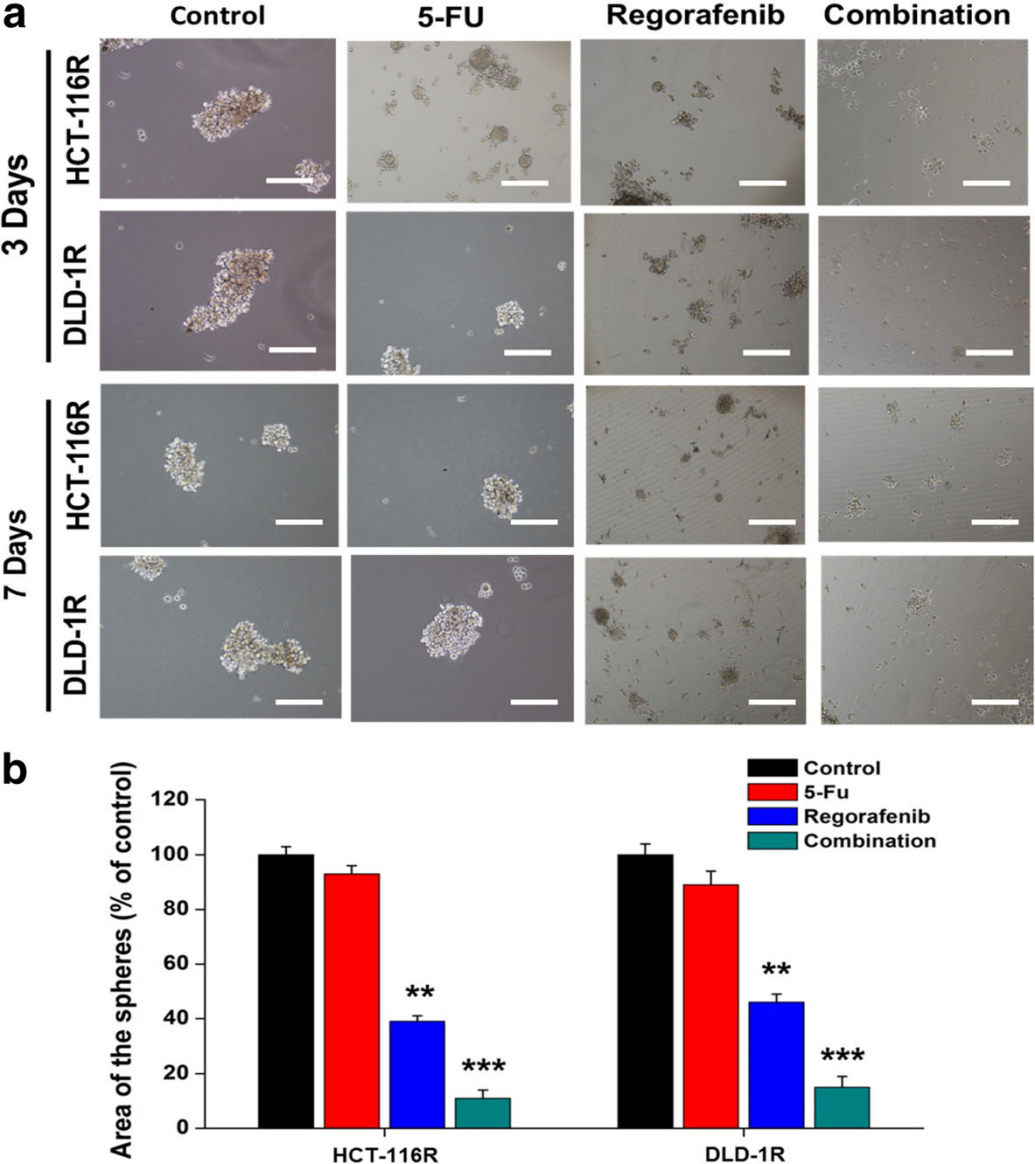
Inhibitory effect of regorafenib and/or 5-FU on tumor sphere generation of DLD-1R and HCT-116R cells. Colonospheres were treated for three or seven days with complete CSCs medium containing DMSO (control), or 10 μM rogozafenib, or 10 μM 5-FU, alone or in combination. **a** Representative cell micrographs after three or seven days of treatment are shown. The area of the culture plates occupied by the spheres (**b**) were scored after three or seven days of treatment. The counts represent the mean values SEM of three independent experiments. The significance of differences was determined with a one-way ANOVA with Bonferroni post-test: ** *p*,0.05, *** *p*,0.001 vs. control

Subsequently, we examined the potential effect of combining regorafenib with 5-FU in treating 5-FU resistant colon cancer cells. Regorafenib alone significantly suppressed the generation of colon tumor spheres in a time-dependent manner (Fig. 5a); 5-FU also exhibited a sight inhibitory effect on the tumor sphere generation as compared to those in the control (untreated) cells. The combination of regorafenib and 5-FU exerted the most significant effect on suppressing the generation of colon tumor spheres in both cell lines. The quantitative analysis of the size of the tumor spheres affected by the treatments was tabulated in Fig. 5b where the average surface area of the tumor spheres was measured and compared.

### Preclinical examination of the combination of regorafenib and 5-FU in suppressing colon tumorigenesis and stemness

**Fig. 6:**
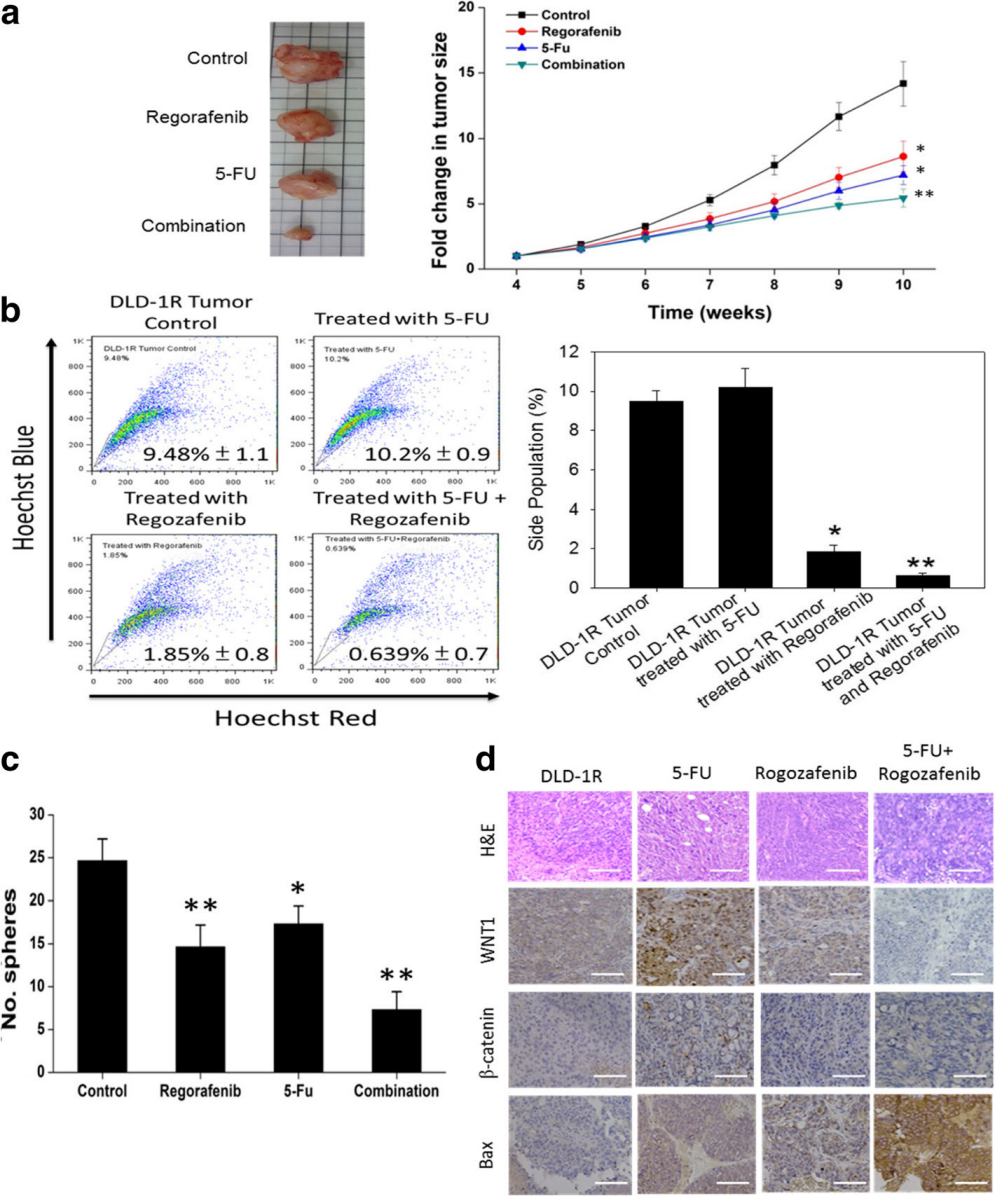
In vivo evaluation of the combination of regorafenib and 5-FU as a therapeutic combination for drug resistant CRC. **a** The tumorigenesis of DLD-1R-inoculated mice with different treatment groups was tracked over time. The fold change of tumor volume was recorded over time. The curve demonstrated that the combination suppressed the tumorigenesis the most followed by 5-FU and regorafenib. The insert shows the photographs of tumor samples harvested after the experiment. **b** Flow cytometry analysis demonstrated that the combination of Regorafenib and 5-FU suppressed the percentage of SP cells most significantly followed regorafenib and 5-FU. **c** Comparative tumor sphere forming ability. DLD-1R tumor cells harvested from the combination treatment showed the lowest tumor sphere generating ability followed by regorafenib and 5-FU. **d** Immunohistochemical analysis of tumor sections. Tumor collected from the combination group showed the lowest staining for β-catenin, WNT1 and ABCG2 while the strongest signal of Bax. *p < 0.05, **p < 0.01

After establishing regorafenib’s inhibitory functions on 5-FU resistant colon cancer cell lines in vitro, we validated our hypothesis in vivo. Immune compromised mice were inoculated subcutaneously with DLD-1R sphere cells (5000 cells per injection). Mice were divided into 4 groups: control (sham injection), regorafenib (10 mg/kg, 5 times/week, i.p.), 5-FU (25 mg/kg, 2 times/week, i.p.). The tumor growth was monitored over time and we found that regorafenib and 5-FU combination had the most significant inhibitory effect followed by 5-FU alone and regorafenib alone (Fig. 6a). Tumor samples were then collected and assayed for tumor sphere generating ability. The tumor cells from the combined treatment group contained approximately 0.639% of SP cells, followed by 1.85% in Regorafenib group, and 10.2% in 5-FU group (Fig. 6b). In support, when the cells were analyzed for their side-population, a similar observation was made in agreement with the final tumor sizes, the tumor cells from combined treatment showed the lowest ability to form tumor spheres followed by regorafenib and 5-FU (Fig. 6c). Using immunohistochemical analysis of the tumor sections, we determined that the expression of ABCG2 (drug resistance marker), β-catenin and WNT1 (stemness markers) were all significantly decreased in the combined treatment group while pro-apoptotic marker, Bax was increased (Fig. 6d).

## Discussion

Regorafenib is a multiple kinase inhibitor which has been attributed to the survival benefits in metastatic colorectal cancer which has failed to respond to all other therapeutics [8]. Regorafenib has been established as a specific inhibitor for receptor kinases including VEGFRs, FGFR and PDGFR [9]. However, resistance against chemotherapeutics represents one of the key features of the presence of cancer stem-like cells. Based on this rationale, we set out to investigate the potential inhibitory effect of Regorafenib on colon cancer stem cells. We first established 5-FU resistant colon cancer cell lines by acclimatizing them under constant, low-dosage of 5-FU. We believe that these cells mimic the residual colon cancer cells post chemotherapy. In support, both HCT-116R and DLD-1R cells showed a significantly increased resistance as compared to their naïve parental counterparts. Consistently, both cells also demonstrated several cancer stem-like features including the increased percentage of CD44+ and side-population cells as well as the enhanced ability to generate tumor spheres. In support, studies have demonstrated that CD44+ cancer stem-like cells obtained from different cancer types, are found to be resistant against drug treatment and with increased expression of powerful oncogenes such as c-Myc and Wnt/β-catenin [10–12] and ones identified in this study, NF-κB, Notch1 and decreased pro-apoptotic marker Bax [13, 14]. More importantly, our 5-FU acclimatized DLD-1R cells showed enhanced tumorigenic ability as compared to their parental counterparts. This is in agreement with the notion where chemotherapy induced drug resistance observed in different cancer types including colon cancer [2, 15, 16]. Even with the targeted therapeutic agent such as cetuximab or panitumumab, resistance has been reported in patients with metastatic colon cancer [17] and has been implicated as a selection process for cancer stem-like cells [18].

In this study, DLD-1R and HCT-116R cells appeared to be sensitive towards regorafenib treatment as demonstrated by the decreased number of colonies, tumor spheres and stem-like phenotypes (tumor spheres and side-populations) and markers (WNT1, mTOR/STAT3, Notch1). Our observation may explain partially why patients with metastatic and drug-resistant colon cancer responded towards the treatment of regorafenib [19]. It is interesting to note that Notch1 expression was linked to the poor prognosis in patients with metastatic colon cancer treated with bevacizumab [20]. According to our results, regorafenib treatment not only resulted in the suppression of Notch1 but also WNT1, CD44, and c-Myc in both DLD-1R and HCT-116R cells.

Mechanistically, regorafenib-treated colon cells (HCT-116R and DLD-1R) showed an elevated level of a various tumor suppressor microRNAs including miR-22, miR-98 and miR-34a while miR-34a as the one being elevated most prominently. The role of miR-34 has been previously linked to the inhibition of tumorigenic phenotypes in HCT-116 cells such as proliferation and metastasis [21]. In addition, an increased level of miR-34a was shown to inhibit the metastatic potential via the suppression of MET signaling pathway in gastric cancer cells [22]. Notably, downstream of MET signaling is STAT3 and CD44 which can be both suppressed by the treatment of regorafenib in our study. In addition, HCT-116R and DLD-1R cells transfected with exogenous miR-34a mimic molecules showed a significantly lower ability to form tumor spheres, linking the role of miR-34a to the generation and maintenance of cancer stem-like cells. Previous reports showed that the increased level of miR-34a was linked to the suppression of the generation of cancer stem cells in prostate and pancreatic cancer via directly down-regulating CD44 expression [23–25], in agreement with our observation in colon cancer cells. In addition, Wnt and β-catenin pathway was shown to be one of the targets for miR-34a in breast cancer, supporting our observations in HCT-116R and DLD-1R 5-FU resistant colon cancer cells.

We also examined the potential of combining regorafenib with 5-FU to overcome drug resistance using DLD-1R and HCT-116R cells. We found that the sequential treatment of regorafenib followed by 5-FU synergistically suppressed the viability of both DLD-1R and HCT-116R cells. It is plausible that the initial treatment of Regorafenib suppressed key stemness markers such as CD44, Wnt1/β-catenin, rendering HCT-116R and DLD-1R cells more sensitive towards the subsequent 5-FU treatment. Our in vitro results were supported by our in vivo mouse experiments where the combination of regorafenib and 5-FU provided the most significant tumor inhibitory effect. The immunohistochemical analysis of the tumor samples confirmed our hypothesis that the combination treatment suppressed the WNT and β-catenin signaling, in addition of ABCG2 and increased Bax expression.

Indeed, the clinical application and/or efficacy of combination of regorafenib and 5FU is very important. In fact, there are many clinical trials being conducted or have been completed. For instance, there is one ongoing phase II clinical trial examining of Regorafenib PO plus 5-FU/LV infusion in 15 mCRC patients who progressed on prior Regorafenib monotherapy as well as 5-FU containing chemotherapy combinations. (https://clinicaltrials.gov/ct2/show/NCT03099486?term=regorafenib%2C+5FU&cond=Colon+Cancer&rank=1). More importantly, in our study, we have provided the potential benefits of using this combination for treating drug-resistant patients through the suppression of cancer stem cells.

## Conclusion

To our knowledge, this is the first report to link regorafenib treatment to the suppression of colon cancer stem cells via the induction of miR-34a level. Our findings may promote the clinical use of the sequential regimen of Regorafenib and 5-FU for treating drug-resistant colon cancer patients.

## Additional file

Additional file 1: **Figure S1.** The increased miR-34a level is associated with the decreased sphere-forming ability. (A) The number of tumor spheres from both HCT-116R and DLD-1R cells were counted under different treatment conditions. Scramble, miR-34a mimic molecule and miR-34a inhibitor. *p < 0.05; **p < 0.01. (B) Western blot analysis of tumor spheres of both HCT-116R and DLD-1R treated with miR-34a mimic and inhibitor molecules. Increased miR-34a by the mimic treatment resulted in the decreased stemness markers, Notch1, WNT1 and oncogene c-Myc. The reversal effect was observed when miR-34a inhibitor was added. (DOCX 313 kb)

## Abbreviations

5-FU: Fluorouracil
ABCG2: ATP-binding cassette transporters
CRC: Colorectal cancer
CSCs: Cancer stem-like cells
DLD-1R: DLD-1 5-FU resistant colon cancer cells
FGFR: Fibroblast growth factor receptor
H&E: Hematoxylin and eosin
HCT-116R: HCT-116 5-FU resistant colon cancer cells
PDGFR: Platelet-derived growth factor receptors
SP: Side population
TCA: Trichloroacetic acid
VEGFRs: Vascular endothelial growth factor

## References

[1] Brenner H, Kloor M, Pox CP (2014). Colorectal cancer. Lancet (London, England).

[2] Lu CS, Shieh GS, Wang CT, Su BH, Su YC, Chen YC, Su WC, Wu P, Yang WH, Shiau AL (2017). Chemotherapeutics-induced Oct4 expression contributes to drug resistance and tumor recurrence in bladder cancer. Oncotarget.

[3] Bertocchi P, Aroldi F, Prochilo T, Meriggi F, Beretta GD, Zaniboni A (2017). Chemotherapy rechallenge after regorafenib treatment in metastatic colorectal cancer: still hope after the last hope?. Journal of chemotherapy (Florence, Italy).

[4] Wilhelm SM, Dumas J, Adnane L, Lynch M, Carter CA, Schutz G, Thierauch KH, Zopf D (2011). Regorafenib (BAY 73-4506): a new oral multikinase inhibitor of angiogenic, stromal and oncogenic receptor tyrosine kinases with potent preclinical antitumor activity. Int J Cancer.

[5] Li J, Qin S, Xu R, Yau TC, Ma B, Pan H, Xu J, Bai Y, Chi Y, Wang L (2015). Regorafenib plus best supportive care versus placebo plus best supportive care in Asian patients with previously treated metastatic colorectal cancer (CONCUR): a randomised, double-blind, placebo-controlled, phase 3 trial. The Lancet Oncology.

[6] Zhang H, Li N, Zhang J, Jin F, Shan M, Qin J, Wang Y (2016). The influence of miR-34a expression on stemness and cytotoxic susceptibility of breast cancer stem cells. Cancer Biol Ther.

[7] Petriz J: Flow cytometry of the side population (SP). Current protocols in cytometry 2007, Chapter 9:Unit9 23. 10.1002/0471142956.cy0923s39 18770857

[8] Grothey A, Van Cutsem E, Sobrero A, Siena S, Falcone A, Ychou M, Humblet Y, Bouche O, Mineur L, Barone C (2013). Regorafenib monotherapy for previously treated metastatic colorectal cancer (CORRECT): an international, multicentre, randomised, placebo-controlled, phase 3 trial. Lancet (London, England).

[9] Fan LC, Teng HW, Shiau CW, Tai WT, Hung MH, Yang SH, Jiang JK, Chen KF (2016). Regorafenib (Stivarga) pharmacologically targets epithelial-mesenchymal transition in colorectal cancer. Oncotarget.

[10] Yoshida GJ, Fuchimoto Y, Osumi T, Shimada H, Hosaka S, Morioka H, Mukai M, Masugi Y, Sakamoto M, Kuroda T (2012). Li-Fraumeni syndrome with simultaneous osteosarcoma and liver cancer: increased expression of a CD44 variant isoform after chemotherapy. BMC Cancer.

[11] Yoshida GJ, Saya H (2014). Inversed relationship between CD44 variant and c-Myc due to oxidative stress-induced canonical Wnt activation. Biochem Biophys Res Commun.

[12] Yoshida GJ, Saya H (2016). Therapeutic strategies targeting cancer stem cells. Cancer Sci.

[13] Pryczynicz A, Gryko M, Niewiarowska K, Cepowicz D, Ustymowicz M, Kemona A, Guzinska-Ustymowicz K (2014). Bax protein may influence the invasion of colorectal cancer. World J Gastroenterol.

[14] Nasu Y, Benke A, Arakawa S, Yoshida GJ, Kawamura G, Manley S, Shimizu S, Ozawa T (2016). In situ characterization of Bak clusters responsible for cell death using single molecule localization microscopy. Sci Rep.

[15] Ma L, Liu T, Jin Y, Wei J, Yang Y, Zhang H (2016). ABCG2 is required for self-renewal and chemoresistance of CD133-positive human colorectal cancer cells. Tumour biology : the journal of the International Society for Oncodevelopmental Biology and Medicine.

[16] Mihalcea DJ, Florescu M, Vinereanu D (2017). Mechanisms and genetic susceptibility of chemotherapy-induced cardiotoxicity in patients with breast Cancer. Am J Ther.

[17] Hsu HC, Thiam TK, Lu YJ, Yeh CY, Tsai WS, You JF, Hung HY, Tsai CN, Hsu A, Chen HC (2016). Mutations of KRAS/NRAS/BRAF predict cetuximab resistance in metastatic colorectal cancer patients. Oncotarget.

[18] Lu H, Chen I, Shimoda LA, Park Y, Zhang C, Tran L, Zhang H, Semenza GL (2017). Chemotherapy-induced Ca2+ release stimulates breast Cancer stem cell enrichment. Cell Rep.

[19] Shirley M, Keating GM (2015). Regorafenib: a review of its use in patients with advanced gastrointestinal stromal Tumours. Drugs.

[20] Paiva TF, de Jesus VH, Marques RA, da Costa AA, de Macedo MP, Peresi PM, Damascena A, Rossi BM, Begnami MD, de Lima VC (2015). Angiogenesis-related protein expression in bevacizumab-treated metastatic colorectal cancer: NOTCH1 detrimental to overall survival. BMC Cancer.

[21] Li C, Lu S, Wang Y, Guo S, Zhao T, Wang X, Song B (2017). Influence of microRNA34a on proliferation, invasion and metastasis of HCT116 cells. Mol Med Rep.

[22] Wei B, Huang QY, Huang SR, Mai W, Zhong XG (2015). MicroRNA34a attenuates the proliferation, invasion and metastasis of gastric cancer cells via downregulation of MET. Mol Med Rep.

[23] Li J, Lam M (2015). Registered report: the microRNA miR-34a inhibits prostate cancer stem cells and metastasis by directly repressing CD44. eLife.

[24] Liu C, Kelnar K, Liu B, Chen X, Calhoun-Davis T, Li H, Patrawala L, Yan H, Jeter C, Honorio S (2011). The microRNA miR-34a inhibits prostate cancer stem cells and metastasis by directly repressing CD44. Nat Med.

[25] Nalls D, Tang SN, Rodova M, Srivastava RK, Shankar S (2011). Targeting epigenetic regulation of miR-34a for treatment of pancreatic cancer by inhibition of pancreatic cancer stem cells. PLoS One.

