# Supplementary figures and images for: Regorafenib suppresses colon tumorigenesis and the generation of drug resistant cancer stem-like cells via modulation of miR-34a associated signaling
Source: J Exp Clin Cancer Res. 2018 Jul 13;37:151. doi: 10.1186/s13046-018-0836-x (PMC6045878; doi:10.1186/s13046-018-0836-x)

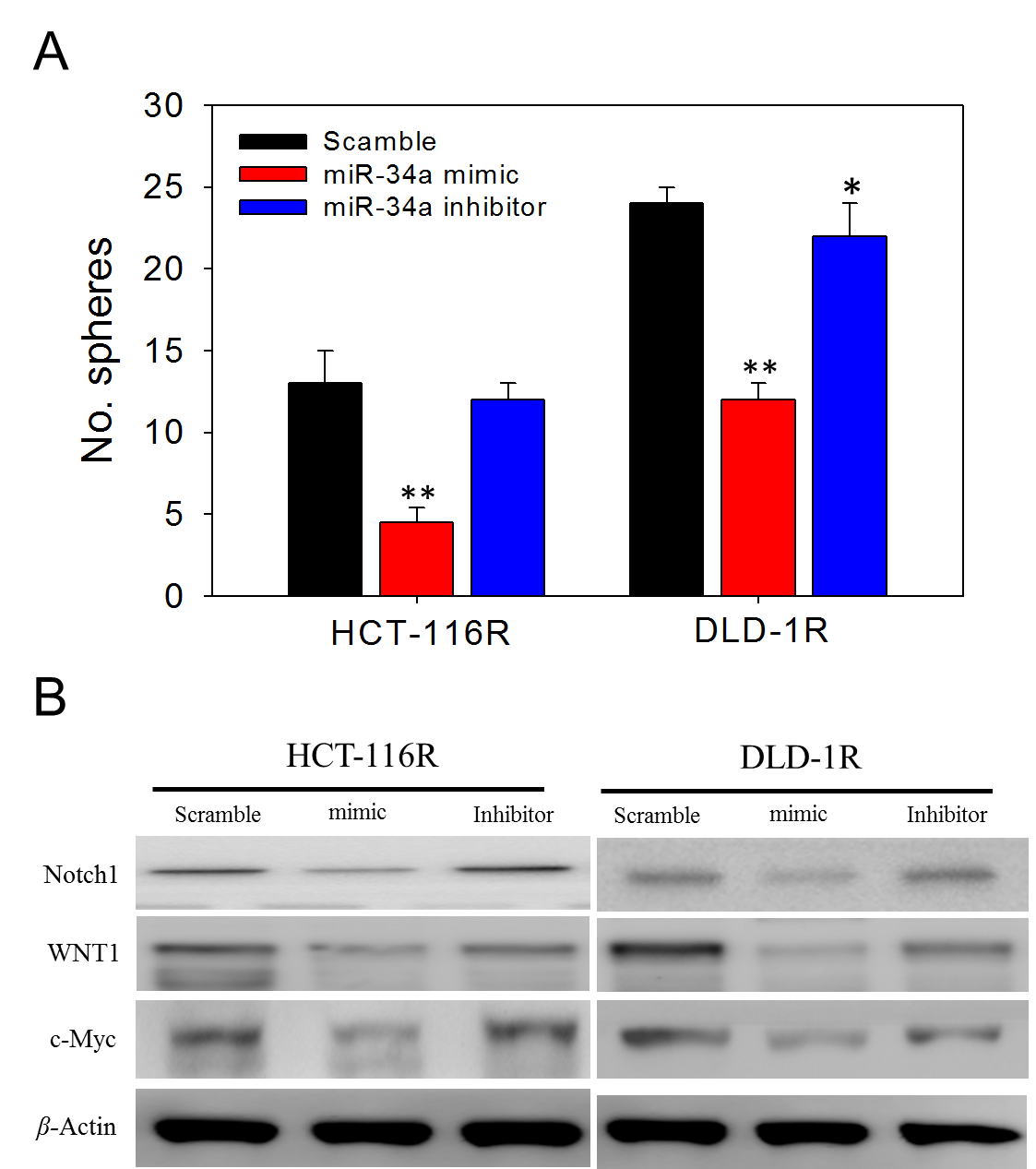


**Figure S1**

Supplement: Supplementary file 1 — Figure S1. The increased miR-34a level is associated with the decreased sphere-forming ability. (A) The number of tumor spheres from both HCT-116R and DLD-1R cells were counted under different treatment conditions. Scramble, miR-34a mimic molecule and miR-34a inhibitor. *p < 0.05; **p < 0.01. (B) Western blot analysis of tumor spheres of both HCT-116R and DLD-1R treated with miR-34a mimic and inhibitor molecules. Increased miR-34a by the mimic treatment resulted in the decreased stemness markers, Notch1, WNT1 and oncogene c-Myc. The reversal effect was observed when miR-34a inhibitor was added. (DOCX 313 kb) [file 13046_2018_836_MOESM1_ESM.docx]
